# Increased expression of stemness markers and altered tumor stroma in hepatocellular carcinoma under TACE-induced hypoxia: A biopsy and resection matched study

**DOI:** 10.18632/oncotarget.22078

**Published:** 2017-10-26

**Authors:** Ji Hae Nahm, Hyungjin Rhee, Haeryoung Kim, Jeong Eun Yoo, Jee San Lee, Youngsic Jeon, Gi Hong Choi, Young Nyun Park

**Affiliations:** ^1^ Department of Pathology, Yonsei University College of Medicine, Seoul, Korea; ^2^ Department of Radiology, Yonsei University College of Medicine, Seoul, Korea; ^3^ Brain Korea 21 PLUS Project for Medical Science, Yonsei University College of Medicine, Seoul, Korea; ^4^ Integrated Genomic Research Center for Metabolic Regulation, Yonsei University College of Medicine, Seoul, Korea; ^5^ Department of Pathology, Seoul National University Hospital, Seoul National University College of Medicine, Seoul, Korea; ^6^ Departments of General Surgery, Yonsei University College of Medicine, Seoul, Korea; ^7^ Severance Biomedical Science Institute, Yonsei University College of Medicine, Seoul, Korea

**Keywords:** hepatocellular carcinoma, stemness, tumor stroma, transarterial chemoembolization, biopsy

## Abstract

**Background:**

Hepatocellular carcinomas (HCCs) expressing stemness markers are characterized by an aggressive behavior, which might be promoted by an altered tumor stroma. Transarterial chemoembolization (TACE) induces severe hypoxia, and its effect on stemness and tumor stroma of HCCs remains unclear. The purpose of this study was to evaluate the sequential changes of stemness and tumor stroma under TACE-induced hypoxia using biopsy and resection-matched HCCs.

**Methods:**

Forty-six biopsy and resection matched HCCs including 10 cases with and 36 cases without preoperative TACE were selected. Immunohistochemistry for stemness (keratin 19 [K19], epithelial cell adhesion molecule [EpCAM], and CD133), hypoxia (carbonic anhydrase IX [CAIX] and vascular endothelial growth factor [VEGF]), and tumor stromal (α-smooth muscle actin [α-SMA] and fibroblast activation protein [FAP]) markers were performed and compared in matched biopsied and resected HCCs with and without TACE.

**Results:**

The accuracy of K19, EpCAM, CD133, CAIX, VEGF, α-SMA and FAP detected on biopsied HCCs was 64% ∼ 86%, using the expression status in resected HCCs as a reference standard in non-TACE group. The sequential change of hypoxia, stemness and stromal marker expression in matched biopsied and resected HCC was greater in TACE group than in non-TACE group (*P* < 0.05 for all). The degree of stemness marker expression was well correlated with those of tumor stromal markers, and the degree of CAIX expression was well correlated with that of K19 (*P* < 0.05).

**Conclusions:**

Stemness marker expression is considered to be increased along with tumor stromal alteration under TACE-induced hypoxia, which might promote the aggressive biology of HCC.

## INTRODUCTION

Hepatocellular carcinoma (HCC) is the seventh most common malignancy worldwide, and the third greatest cause of cancer related mortality, especially in Asia and sub-Saharan Africa [1, 2]. Transarterial chemoembolization (TACE) is a popular loco-regional therapy in downstaging or bridging to make curative treatments (e.g. resection, transplantation) of HCC possible [3]. Although TACE induces marked tumor necrosis by obstructing tumor-feeding arteries with chemotherapy emulsioned with lipiodol and embolic agents [4, 5], a significant number (27 ∼ 72%) of HCCs show residual viable tumor after TACE [6].

Cancer stem cells (CSCs), characterized by their ability to self-renew and propagate tumors, play an important role in tumor maintenance and recurrence [7, 8]. HCCs expressing stemness-related markers, such as keratin 19 (K19), epithelial cell adhesion molecule (EpCAM), or CD133, are reportedly associated with an aggressive biological behavior with poorer prognosis, compared to HCCs not expressing these markers [9–11]. Hypoxia has been found to be involved in maintenance of CSCs of several cancers, including HCC, glioblastoma, breast cancer, and hematologic malignancies [12–15]. Recently, increased expression of stemness-related markers was reported in resected/explanted HCCs after TACE treatment [16, 17].

The biological behavior of tumors is reportedly determined by not only tumoral epithelial cells themselves but also by the tumor stroma, which is a complicated system composed of extracellular matrix proteins, proteolytic enzymes, blood vessels, and a variety of cellular components, including cancer-associated fibroblasts (CAFs) and immune cells, etc [18–21]. CAFs, histologically categorized as myofibroblasts or activated fibroblasts, were also reported to be associated with aggressive biological behavior, poor prognosis, and resistance to chemotherapy and radiation therapy in several tumors including HCC [22–25]. HCCs usually contain no or only a little amount of fibrous stroma; nevertheless, we previously reported that HCCs with stemness markers showed tumor stroma [9]. Interestingly, explanted HCCs after TACE were reported to be more fibrotic than those without [16].

Therefore, TACE-induced hypoxia might modulate stemness and tumor stroma of HCC, which are associated with poor clinical outcomes. However, the sequential changes in the expression of stemness markers and tumor stroma of HCCs after TACE remain unclear. In this study, we first checked whether the immunoprofiles of biopsied HCCs, which contain only small portions of tumor, can represent those of whole tumor, by comparing the immunoprofiles of stemness (K19, EpCAM, and CD133), hypoxia (carbonic anhydrase IX [CAIX] and vascular endothelial growth factor [VEGF]), and tumor stromal markers (α-smooth muscle actin [α-SMA] and fibroblast activation protein [FAP]) between matched biopsied and resected HCCs in non-TACE group. Then the sequential changes in the expression status of stemness, hypoxia, and tumor stromal markers were evaluated by comparing the change in expression status of these markers in matched preoperative biopsied and resected HCCs, between HCCs with preoperative TACE and those without.

## RESULTS

### Comparison of the immunoprofiles between matched preoperative biopsies and resected HCCs

**Figure 1:**
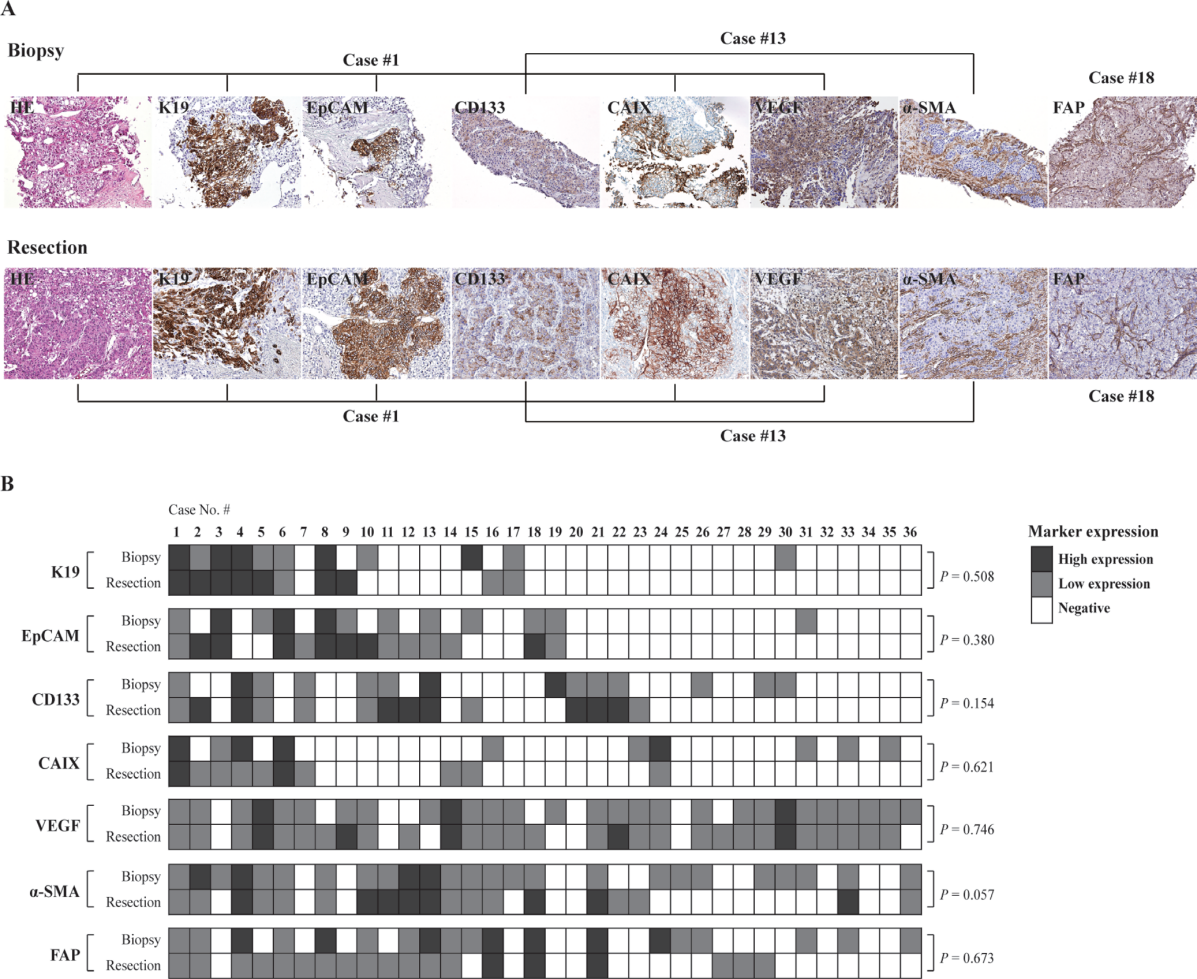
(**A**) Hepatocellular carcinoma showing expression of K19, EpCAM, CD133, CAIX and VEGF in tumoral epithelial cells and expression of α-SMA and FAP in tumoral stromal cells in matched biopsied and resected HCC without preoperative TACE. The photos of H-E, K19, EpCAM, CAIX and VEGF were taken from case number 1, those of CD133 and α-SMA were taken from case number 13, and those of FAP were taken from case number 18. (**B**) A summary of the immunoprofiles in the matched preoperative biopsied and resected HCCs without preoperative TACE. The cases with high expression, low expression, and negative expression are indicated by black, gray and white boxes, respectively.

To investigate whether the immunoprofiles of biopsied HCC tissue may represent those of the whole tumor in resected HCCs, we compared the expression status of stemness-, hypoxia- and stromal- markers between matched preoperative baseline biopsies and the resected HCCs in the non-TACE group (*n =* 36) (Figure 1). Only the non-TACE group was analyzed in order to exclude the possible effects of preoperative TACE on the immunoprofiles of resected HCCs. No significant differences were found in the degrees of K19, EpCAM, CD133, CAIX, VEGF, α-SMA and FAP expression between biopsied and resected HCC tissues (Figure 1B).

In addition, the diagnostic performance of immunohistochemical result based on biopsied HCCs was evaluated using the immunoprofiles of resected HCCs as a standard of reference, and the expression status of each marker was checked as binary classification (i.e. negative and positive). The accuracy of immunohistochemical stains on biopsies was 86%, 78% and 78% for K19, EpCAM, and CD133, respectively, and 72%, 81%, 72% and 64% for CAIX, VEGF, α-SMA and FAP, respectively (Table 1). Taken together, the immunoprofiles of biopsied HCCs may represent those of resected HCCs.

**Table 1:** Sensitivity, specificity, and accuracy of immunoexpression detected in the baseline preoperative biopsied HCCs using the immunoprofiles of resected HCCs as standard of references in non-TACE group (*n* = 36)

Immunomarkers	Sensitivity	Specificity	Accuracy
K19	80%	88%	86%
EpCAM	64%	86%	78%
CD133	71%	82%	78%
CAIX	50%	81%	72%
VEGF	86%	57%	81%
α-SMA	89%	53%	72%
FAP	65%	63%	64%

Abbreviations: K19, keratin 19; EpCAM, epithelial cell adhesion molecule; CAIX, carbonic anhydrase IX; VEGF, vascular endothelial growth factor; α-SMA, α-smooth muscle actin; FAP, fibroblast activation protein.

### The sequential change in the expression of stemness, hypoxia and stromal markers after TACE

No significant differences were seen in the expression status of K19, EpCAM, CD133, CAIX, VEGF, α-SMA and FAP in the preoperative baseline biopsied HCCs between the TACE and non-TACE groups. In contrast, for resected HCCs, the incidence of EpCAM and α-SMA expression was significantly higher in HCCs with preoperative TACE than in those without (*P* < 0.05 for both). The clinicopathological features of resected HCCs with and without preoperative TACE are summarized in Table 2. The resected HCCs with preoperative TACE showed younger age, poorer differentiation of HCC, higher incidence and greater extents of tumor necrosis, compared to those without preoperative TACE (*P* < 0.05 for all). Other clinicopathological features of resected HCCs, including TNM stage showed no significant difference between the two groups.

**Table 2:** Clinicopathological features of HCCs in the TACE group and non-TACE group

Clinicopathological features	TACE group (*n* = 10)	non-TACE group (*n* = 36)	*P* value
Age (year, median, IQR)	52.0 (27.5 – 56.5)	61.5 (54.0 – 68.8)	0.033^*^
Gender (male/female, %)	9 (90%)/1 (10%)	32 (89%)/4 (11%)	>0.999
Etiology (HBV/HCV/Alcohol/Unknown, %)	8 (80%)/0 (0%)/0 (0%)/ 2 (20%)	23 (64%)/1 (3%)/7 (19%)/ 5 (14%)	0.438
Cirrhosis (%)	5 (50%)	10 (28%)	0.257
Serum AST (IU/L, median, IQR)	61.0 (33.0 – 105.0)	31.0 (22.3 – 35.0)	0.298
Serum ALT (IU/L, median, IQR)	27.0 (15.0 – 50.0)	28 (18.3 – 47.5)	0.723
Serum alpha-fetoprotein (IU/mL, median, IQR)	299.8 (39.1 – 43872.5)	9.4 (3.3 – 35.4)	0.200
Serum PIVKA-II (AU/mL, median, IQR)	329 (643.0 – 1606.0)	38.5 (20.3 – 1211.3)	0.066
**Tumor pathology in resected specimens**			
Tumor number (one / two / three, %)	10 (100%)/0 (0%)/0 (0%)	31 (86%)/4 (11%)/1 (3%)	0.459
Diameter of entire tumor (cm, median, IQR)	7.0 (5.0 – 11.5)	4.0 (3.0 – 6.8)	0.134
Viable tumor area (cm^2^, median, range)	2.6 (0.8 – 27.0)	8.8 (1.2 – 110.4)	0.003^*^
Differentiation (Edmonson-Steiner Grade I/II/III, %)	2 (20%)/1 (10%)/7 (70%)	4 (11%)/23 (64%)/9 (25%)	0.009^*^
Presence of tumor necrosis (%)	10 (100%)	20 (56%)	0.002^*^
Proportion of tumor necrosis area to entire tumor region (%, median, IQR)	80.0 (67.5 – 95.0)	0 (0 – 10.0)	<0.001^*^
Microvascular invasion (%)	8 (70%)	15 (42%)	0.071
Tumor capsule formation (%)	7 (70%)	22 (61%)	0.723
Serosal invasion (%)	7 (70%)	24 (67%)	>0.999
TNM stage (stage I / II / III, %)	2 (20%)/8 (80%)/0 (0%)	19 (53%)/16 (44%)/1 (3%)	0.134
**Immunomarker expression in biopsied specimens**			
K19	5 (50%)	11 (31%)	0.283
EpCAM	6 (60%)	12 (33%)	0.157
CD133	3 (30%)	9 (25%)	0.706
CAIX	3 (30%)	10 (28%)	>0.999
VEGF	7 (70%)	28 (78%)	0.682
α-SMA	6 (60%)	25 (69%)	0.573
FAP	5 (50%)	19 (53%)	0.876
**Immunomarker expression in resected specimens**			
K19	5 (50%)	10 (28%)	0.257
EpCAM	8 (80%)	14 (39%)	0.032^*^
CD133	6 (60%)	13 (36%)	0.277
CAIX	6 (60%)	10 (30%)	0.074
VEGF	6 (60%)	29 (81%)	0.220
α-SMA	9 (90%)	15 (42%)	0.011^*^
FAP	9 (90%)	19 (53%)	0.064

Abbreviations: HCC, hepatocellular carcinoma; TACE, transarterial chemoembolization; IQR, interquartile range; HBV, hepatitis B virus; HCV, hepatitis C virus; AST, aspartate aminotransferase; ALT, alanine aminotransferase; PIVKA-II, protein induced by vitamin K absence or antagonist II; TNM, tumor-node-metastasis; K19, keratin 19; EpCAM, epithelial cell adhesion molecule; CAIX, carbonic anhydrase IX; VEGF, vascular endothelial growth factor; α-SMA, α-smooth muscle actin; FAP, fibroblast activation protein. ^*^P < 0.05

**Figure 2:**
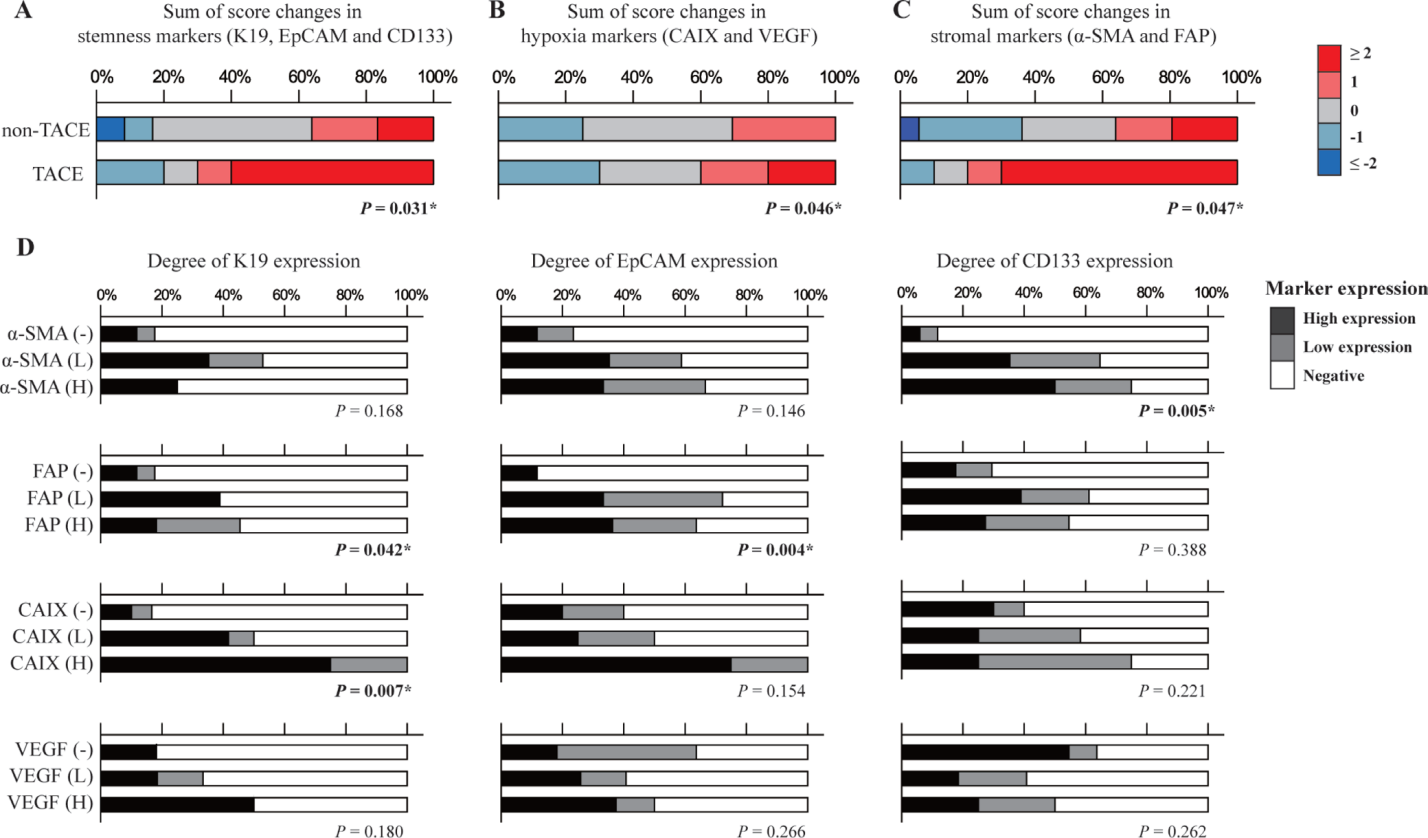
Sum of score changes of (**A**) stemness markers (K19, EpCAM and CD133), (**B**) hypoxia markers (CAIX and VEGF), and (**C**) stromal markers (α-SMA and FAP) checked in matched biopsied and resected HCC are compared between HCCs with preoperative TACE and those without TACE. The red bar represents that the expression of markers is higher in resected HCCs compared biopsied HCCs, in contrast that the blue bar represents decreased expression in resected HCCs than in biopsied HCCs. Deeper color indicates greater change, and the grey bar represents no change. (**D**) The correlation between expression of stemness markers (K19, EpCAM, and CD133) and those of stromal markers (α-SMA and FAP) and hypoxia markers (CAIX and VEGF). The black, grey, and white bars represent high, low, and no expression, respectively. ^*^P < 0.05

The sequential change in the degree of immunoexpression between biopsied and resected HCCs was evaluated and compared between HCCs with preoperative TACE and those without. The sum of score change in three stemness markers (K19, EpCAM, and CD133) showed a significant difference between HCCs with preoperative TACE and those without (*P* = 0.031), indicating an increased expression of stemness markers after TACE (Figure 2A). The sum of score change in hypoxia markers (CAIX and VEGF) showed a significant difference between HCCs with preoperative TACE and those without (*P* = 0.046), indicating an increased hypoxic tumor microenvironment after TACE (Figure 2B). Similarly, the sum of score change in stromal markers (α-SMA and FAP) showed a significant difference between HCCs with preoperative TACE and those without (*P* = 0.047), suggesting an altered tumor stroma after TACE (Figure 2C).

The relationship of stemness marker expression with those for tumor stroma and hypoxia was evaluated in all resected HCCs including TACE and non-TACE groups (*n =* 46) (Figure 2D). The degree of K19 and EpCAM expression was well correlated with tumor stromal FAP expression (*P* = 0.042 and *P* = 0.004, respectively), and the degree of CD133 expression was well correlated with tumor stromal α-SMA expression (*P* = 0.005). The degree of CAIX expression was well correlated with that of K19 expression (*P* = 0.007), but not for those of EpCAM and CD133 (*P* > 0.05 for both). The degree of VEGF expression was not correlated with any stemness marker of K19, EpCAM and CD133 (*P* > 0.05 for all).

### The prognostic significance of stemness, hypoxia and stromal marker expression in biopsied and resected HCCs in non-TACE group

**Figure 3:**
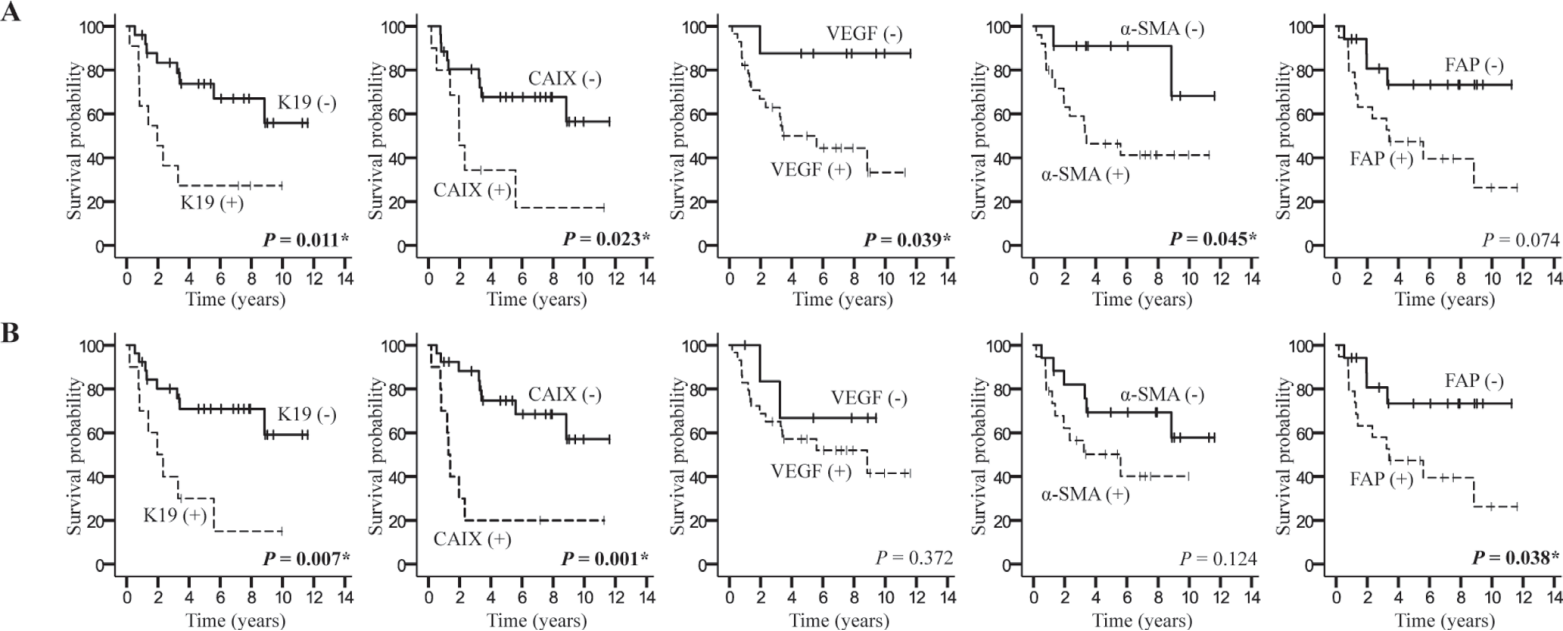
Kaplan-Meier survival curve representing the overall survival of hepatocellular carcinoma patients according to K19, CAIX, VEGF, α-SMA and FAP expression detected in (**A**) biopsied and (**B**) resected HCCs in non-TACE group. Positive expression is indicated by a dotted line, and negative expression is indicated by a solid line. ^*^P < 0.05

The prognostic significance of clinicopathologic features, and expression of stemness, hypoxia and stromal markers was evaluated in non-TACE group to exclude the effect of TACE on HCCs. In the preoperative biopsied HCCs of the non-TACE group, univariate analysis of immunoprofiles performed on biopsied HCCs revealed that expression of K19 (*P* = 0.011), CAIX (*P* = 0.023), VEGF (*P* = 0.039) and α-SMA (*P* = 0.045) were adverse prognostic factors for overall survival (Table 3, Figure 3A). Other immunomarkers (including EpCAM, CD133 and FAP), and other preoperatively collected clinical data (including age, gender, etiology and serum markers) showed no significant impact on overall survival.

**Table 3:** Univariate analysis for overall survival in the non-TACE group HCCs (*n* = 36)

			Overall survival (%)		
		Event / Total number	3-year	5-year	*P* value
**Clinical data collected at the time of biopsy**					
Age	<60 years	8/16	69%	56%	0.885
	≥60 years	8/20	69%	63%	
Gender	Male	14/32	68%	60%	0.980
	Female	2/4	75%	50%	
Etiology (Hepatitis B)	HBV	7/13	78%	69%	0.138
	Others	9/23	52%	42%	
Serum alpha-fetoprotein	<400 IU/mL	12/30	65%	61%	0.535
	≥400 IU/mL	4/6	67%	50%	
Serum PIVKA-II	<400 AU/mL	6/20	79%	73%	0.347
	≥400 AU/mL	4/8	63%	47%	
**Immunoprofiles in biopsied specimens**					
K19	Negative	8/25	79%	74%	0.011^*^
	Positive	8/11	36%	27%	
EpCAM	Negative	10/24	74%	64%	0.667
	Positive	6/12	58%	50%	
CD133	Negative	12/27	65%	56%	0.749
	Positive	4/9	78%	67%	
CAIX	Negative	9/26	80%	68%	0.023^*^
	Positive	7/10	34%	34%	
VEGF	Negative	1/8	86%	86%	0.039^*^
	Positive	15/28	63%	50%	
α-SMA	Negative	2/11	91%	91%	0.045^*^
	Positive	14/25	59%	46%	
FAP	Negative	5/17	82%	75%	0.074
	Positive	11/19	56%	45%	
**Pathological features and immunoprofiles in resected specimens**					
Tumor size	<5cm	7/22	77%	71%	0.112
	≥5cm	9/14	57%	41%	
Tumor multiplicity	Single	13/31	66%	59%	0.765
	Multiple	3/5	80%	53%	
Differentiation (Edmonson-Steiner grade)	Grade I/II	12/27	70%	62%	0.850
	Grade III	4/9	65%	52%	
Microvascular invasion	Negative	6/21	79%	74%	0.024^*^
	Positive	10/15	47%	40%	
TNM stage	Stage I	5/19	83%	70%	0.038^*^
	Stage II/III	11/17	53%	46%	
Cirrhosis	None	12/26	65%	56%	0.671
	Present	4/10	79%	68%	
K19	Negative	8/26	80%	71%	0.007^*^
	Positive	8/10	40%	30%	
EpCAM	Negative	8/22	81%	70%	0.214
	Positive	8/14	50%	43%	
CD133	Negative	10/23	72%	63%	0.568
	Positive	6/13	62%	53%	
CAIX	Negative	8/26	88%	75%	0.001^*^
	Positive	8/10	20%	20%	
VEGF	Negative	2/7	83%	67%	0.372
	Positive	14/29	65%	57%	
α-SMA	Negative	9/21	82%	69%	0.124
	Positive	7/15	56%	50%	
FAP	Negative	4/17	81%	73%	0.038^*^
	Positive	12/19	58%	47%	

Abbreviations: HCC, hepatocellular carcinoma; TACE, transarterial chemoembolization; HBV, hepatitis B virus; PIVKA-II, protein induced by vitamin K absence or antagonist II; K19, keratin 19; EpCAM, epithelial cell adhesion molecule; CAIX, carbonic anhydrase IX; VEGF, vascular endothelial growth factor; α-SMA, α-smooth muscle actin; FAP, fibroblast activation protein. ^*^P < 0.05

The pathological features of resected non-TACE group HCCs were also evaluated for the overall survival, and the expression of K19, CAIX and FAP, and microvascular invasion, and TNM stage were significant for poor overall survival (*P* < 0.05 for all). (Table 3, Figure 3B). Multivariate analysis for overall survival was performed using five postoperatively collected factors (K19, CAIX, and FAP expression, microvascular invasion and TNM stage). CAIX and FAP expression showed statistical significance (*P* = 0.001 and *P* = 0.022, respectively) for overall survival (Table 4).

**Table 4:** Multivariate analysis for overall survival in the resected HCCs of non-TACE group (*n* = 36)

Variables	Multivariate analysis	
	Hazard ratio (95% CI)	*P* value
CAIX (positive)	6.3 (2.1 – 19.2)	0.001^*^
FAP (positive)	4.1 (1.2 – 13.6)	0.022^*^

Abbreviations: HCC, hepatocellular carcinoma; TACE, transarterial chemoembolization; CAIX, carbonic anhydrase IX; FAP, fibroblast activation protein. ^*^P < 0.05

## DISCUSSION

Hypoxia can modulate tumor biology by activation of hypoxia adaptation pathways, especially hypoxia inducible factor-1 (HIF-1) [3, 12, 26]. CAIX, a direct transcriptional target of HIF-1α, is considered an endogenous marker for the transcriptional activity of HIF-1α [27, 28], and can be more easily detected by immunohistochemical stain than HIF-1α, which has a short half-life (<5 min) [29]. VEGF expression is also induced by hypoxia response genes including HIF-1α, and promotes neovascularization [30]. Recently, hypoxia has been reported to be important in reprogramming to cancer stem cell phenotype and maintenance of cancer stem cells in several cancer types, including HCC [12–15, 31, 32], and increased expression of stemness markers was reported in resected/explanted HCCs after TACE treatment [16, 17]. We also reported that the expression of K19, EpCAM and CAIX was significantly higher in residual viable HCCs with preoperative TACE compared to those without, and that K19, EpCAM, and CAIX were more frequently expressed in HCCs with a greater number of TACE sessions, suggesting that evaluation of these markers in biopsied HCC tissue might have an additional value in predicting HCC outcome, especially for TACE-treated cases [33].

To address the question of whether the immunoprofiles of small needle biopsy tissues are representative of the whole tumor, we compared the expression status of stemness-, hypoxia- and tumor stroma-related markers in matched preoperative biopsied and resected HCC tissues without TACE. Using the immunoprofiles of the resected HCCs as a standard of reference, the accuracies of marker expression ranged from 64% to 86%, suggesting that the immunoprofiles of biopsied HCCs could be considered to be representative of whole HCCs. A gene expression analysis study on different areas of the same tumor demonstrated little differences in the gene expression profiles according to the location [34]. In addition, the expression of K19 and CAIX in biopsied HCC tissues as well as their expression in resected HCCs was significant for poor overall survival in this study.

In addition, it remains unclear whether the extent of K19, EpCAM and CAIX expression is constant or changed under TACE-induced hypoxia. To address this question, the sequential change in the degree of immunoexpression between biopsied and resected HCCs was evaluated and compared between HCCs of the TACE and non-TACE groups. Although there was extensive tumor necrosis (up to 95%, median 80%) in HCCs of TACE group, the viable tumor area ranged from 0.8 cm^2^ to 27.0 cm2 (median, 2.6 cm2). Therefore, the immunoprofiles of the viable tumor in TACE group were considered to be representative. While there was no significant difference in stemness-related marker expression status in the baseline preoperative biopsies of both groups, the sum of score change for stemness markers was significantly higher in the TACE group HCCs compared to the non-TACE group. Therefore, it is suggested that stemness marker expression may be increased in HCCs under TACE-induced hypoxic conditions. Taken together, we discerned that HCC tumor cells expressing stemness markers, which are considered as CSCs, may have a survival advantage in hypoxic tumor environment induced by TACE, and that these tumor cells may proliferate under TACE-induced hypoxia, as evidenced by the increased tumor extent of stemness-related marker expression in this study. In addition, the degree of K19 expression was correlated with that of CAIX expression in resected HCCs. Accordingly, CAIX was reported to increase extracellular acidity and result in metabolic reprogramming, and maintenance of CSCs in breast cancer and prostate cancer [14, 35].

We found that the sequential change in sum of hypoxia marker (CAIX and VEGF) expression in matched biopsied and resected HCC was greater in TACE group than in non-TACE group, suggesting that hypoxic condition increased after TACE. However, there were no significant differences in CAIX and VEGF expression between resected HCCs of TACE and non-TACE group. It could be speculated that large sized HCCs in the TACE group had already adapted to the hypoxic condition caused by the imbalance between rapid growth and blood supply before TACE. Accordingly, CD34-positive/VEGF-negative HCCs have been reported to be resistant to TACE-induced hypoxia, as they had already developed a sufficient vascular network without requiring further neoangiogenesis [36].

Abundant intratumoral fibrous stroma is not a typical feature of HCC; we previously reported that HCCs with this feature exhibited high expression of stemness markers with upregulated transforming growth factor-β (TGF-β) signaling and epithelial-mesenchymal transition (EMT) regulators [37]. Recently, tumor stromal cells were reported to promote cancer cells to gain CSC properties through production of IL-6 or TGF-β1-induced EMT [38, 39]. FAP was shown to increase stromal cell proliferation and invasiveness, to reduce apoptosis, and to be associated with worse prognosis in colon cancer and pancreatic cancer [40, 41]. In this study, the expression score difference of stromal markers (α-SMA and FAP) between preoperative biopsied and resected HCCs was significantly higher in TACE group HCCs compared with the non-TACE group. α-SMA expression was also higher in resected TACE group HCCs compared to non-TACE group HCCs, while there was no significant difference in α-SMA expression status in the baseline preoperative biopsied HCCs between two groups. Taken together, TACE-induced hypoxia is considered to alter tumor stroma, which may in turn contribute to the aggressiveness of HCCs.

Crosstalk between the tumoral epithelia and stroma has been shown to facilitate tumor growth and cancer progression through EMT, in which TGF-β/ platelet-derived growth factor signaling plays a crucial role [42]. Interestingly, we found a positive correlation between the expression of stemness markers and stromal markers. Therefore, CSCs are not considered to be a fixed cell population and their plasticity might be regulated by tumor stromal factors, and the altered tumor stroma in hypoxia might provide a niche for CSC to proliferate in the residual HCC after TACE. In fact, CD133 transcription has been reported to be induced by IL-6/STAT3 signaling through functional cooperation with NF-κB and HIF-1α during liver carcinogenesis [43].

This is the first study to comprehensively evaluate the changes in immunoprofiles of tumor cells and tumor stroma after TACE using matched biopsied and resected HCCs. One of the limitations of this study is the small number of biopsy-resection matched cases. It is difficult to procure a large cohort of biopsy-resection matched HCCs, as biopsy confirmation is not recommended for HCC when imaging findings are diagnostic according to the current guidelines. Survival analysis was not conducted separately for the TACE and non-TACE groups, due to the small number of biopsy-resection matched cases. Further studies based on a larger number of cases from multiple centers are required.

In conclusion, the expression of stemness markers is considered to be increased along with alteration of tumor stroma under TACE-induced hypoxia, which might promote the aggressive biology of HCC. Therefore, checking the expression status of these markers in biopsied HCCs may help to predict a poor outcome of HCCs, especially for TACE treatment.

## MATERIAL AND METHODS

### Case selection and clinicopathologic analysis

The study subjects comprised 46 patients who were diagnosed as HCC by preoperative biopsies and underwent subsequent surgical resection at Severance Hospital from January 2001 to February 2014. All preoperative baseline biopsies were taken prior to any treatment. After the biopsy, 10 cases underwent preoperative TACE (“TACE group”) and the remaining 36 cases received no TACE (“non-TACE group”). In all cases with multiple tumors (5 cases in non-TACE group, none in TACE group), the biopsy was performed from the largest nodule. Subsequently, the 46 patients received surgical resection of the HCCs. TACE, containing lipiodol with adriamycin or doxorubicin, was performed once in seven cases, twice in two cases, and three times in one case. The average intervals between biopsy and resection for the non-TACE group and TACE group were 72 days (range, 6 ∼ 1039 days) and 65 days (range, 16 ∼ 247 days), respectively. Cases that underwent other types of treatment, such as radiofrequency ablation or chemotherapy, were excluded, and cases with total necrosis after TACE were also not included in this study, as their tumor immunoprofiles could not be assessed.

Histopathologic analysis was performed for tumor number, diameter of entire tumor, viable tumor area, differentiation (Edmondson-Steiner grade), tumor capsule formation, serosal invasion, microvascular invasion, presence of tumor necrosis, and proportion of tumor necrosis area to entire tumor region. In the cases with multiple tumors, histopathologic analysis was performed from the largest nodule. Tumor-node-metastasis (TNM) classification was analyzed according to the 7th American Joint Committee on Cancer/International Union against Cancer (AJCC/UICC) staging system. Clinical data, including age, sex, etiology, cirrhosis, serum aspartate aminotransferase (AST), serum alanine aminotransferase (ALT), serum alpha-fetoprotein (AFP), and serum protein induced by vitamin K absence or antagonist II (PIVKA-II), and follow up data were obtained from the electronic medical records. The mean follow-up period after resection was 60.5 months (range, 2.5 ∼ 142.4 months). This study was approved by the Institutional Review Board of Severance Hospital, Yonsei University College of Medicine (Seoul, Korea), and the need for patient consent was waived (4–2016–0826).

### Immunohistochemical evaluation

Representative blocks of formalin-fixed paraffin embedded tissues were used for immunohistochemical stains. In the case of multiple nodules, we performed the immunohistochmical staining in the largest nodule, which was matched with biopsied HCC. Details of the antibodies used are summarized in Table 5, and immunohistochemistry was performed as previously described [9]. K19, EpCAM, CD133, CAIX and VEGF were stained in the tumor epithelial cells, whereas α-SMA and FAP were stained in the cancer associated fibroblasts (CAFs) of tumor stroma. The positive expression area was defined as a percentage of the total tumor area, and the intensity of staining was evaluated as follows: 1, weak; 2, moderate; or 3, strong. Final scores were obtained by multiplying the positive expression area (%) by intensity, and divided into negative group (score 0), and positive group of low expression (1+) and high expression (2+). For stemness-related and hypoxia markers (K19, EpCAM, CD133, CAIX and VEGF), negative (0) was defined as scores <1 in biopsied and <5 in resected HCCs, low expression (1+) was defined as scores 1 ∼ 50 in biopsied and 5 ∼ 50 in resected HCCs, and high expression (2+) was defined as score >50 in both biopsies and resection. For stromal markers (α-SMA and FAP), negative (0) was defined as scores <5 in biopsied and <10 in resected HCCs, low expression (1+) was defined as scores 5 ∼ 20 in biopsies and 10 ∼ 20 in resected HCCs, while high expression (2+) was defined as score >20 in both types of specimen.

**Table 5:** Information on primary antibodies

Antibody	Source	Clone	Dilution	Antigen retrieval	Blocking
K19	Dako (Glostrup, Denmark)	RCK108	1:25	Protease K (Dako, Glostrup, Denmark)	-
EpCAM	Calbiochem (Darmstadt, Germany)	VU-1D9	1:3000	Microwave, citrate (pH 6.0)	-
CD133	Miltenyi Biotech (Bergisch Gladbach, Germany)	W6B3C1	1:25	Microwave, citrate (pH 6.0)	2% BSA for 0.5h at RT
CAIX	Abcam (Cambridge, UK)	rabbit polyclonal	1:2000	Microwave, citrate (pH 6.0)	5% BSA for 5h at RT
VEGF	Santa Cruz Biotechnology (Dallas, TX, USA)	C-1	1:25	No treatment	
α-SMA	Dako (Glostrup, Denmark)	1A4	1:1000	No treatment	-
FAP	Vitatex (Stony Brook, NY, USA)	Seprase D8	1:100	Microwave, citrate (pH 6.0)	

Abbreviations: K19, keratin 19; EpCAM, epithelial cell adhesion molecule; CAIX, carbonic anhydrase-IX; VEGF, vascular endothelial growth factor; α-SMA, α-smooth muscle actin; FAP, fibroblast activation protein; BSA, bovine serum albumin; RT, room temperature.

In order to evaluate the sequential change in the expression status of each marker between the biopsies and resected specimens, we calculated the differences between the expression scores of each marker in the biopsies and matched resected specimens (“score change”), and then added the differences of each markers to yield the “sum of score change”. For example, if K19 expression was low (1+) in the biopsy and high (2+) in the matched resected specimen, the score change was 1. In a similar manner, if the same patient showed low EpCAM (1+) and low CD133 (1+) expression in the biopsy, and high EpCAM (2+) and low CD133 (1+) in the resected tumors, the score changes were 1 and 0, respectively. The sum of score change was therefore 2 (1 + 1 + 0). The same method was applied for the hypoxia-related markers and stromal markers. The staining results were assessed by two pathologists unaware of the clinicopathological data for each case (Figure 1A).

### Statistical methods

Statistical analyses were carried out using SPSS software (version 21.0, SPSS Inc., Chicago, Illinois). Chi-square test or Fisher’s exact test was used. Univariate survival analyses were performed by the Kaplan-Meier method with log-rank test, and multivariate survival analyses were conducted by Cox regression with forward conditional method. Statistical significance was assumed when *P* < 0.05.

## Abbreviations

HCC: hepatocellular carcinoma
CSC: cancer stem cell
K19: keratin 19
EpCAM: epithelial cell adhesion molecule
CD133: cluster of differentiation 133
α-SMA: α-smooth muscle actin
FAP: fibroblast activation protein
HIF: hypoxia inducible factor
CAIX: carbonic anhydrase IX
VEGF: vascular endothelial growth factor
H&E: hematoxylin-eosin
TACE: transarterial chemoembolization
AST: aspartate aminotransferase
ALT: alanine aminotransferase
AFP: alpha-fetoprotein
PIVKA-II: protein induced by vitamin K absence or antagonist II

## References

[1] Yang JD, Roberts LR (2010). Hepatocellular carcinoma: A global view. Nat Rev Gastroenterol Hepatol.

[2] Yu SJ (2016). A concise review of updated guidelines regarding the management of hepatocellular carcinoma around the world: 2010–2016. Clin Mol Hepatol.

[3] Harris AL (2002). Hypoxia: a key regulatory factor in tumour growth. Nat Rev Cancer.

[4] Xiao EH, Li JQ, Huang JF (2007). Effect of preoperative transcatheter arterial chemoembolization on proliferation of hepatocellular carcinoma cells. World J Gastroenterol.

[5] European Association For The Study Of The Liver; European Organisation For Research And Treatment Of Cancer (2012). EASL-EORTC clinical practice guidelines: management of hepatocellular carcinoma. J Hepatol.

[6] Chua TC, Liauw W, Saxena A, Chu F, Glenn D, Chai A, Morris DL (2010). Systematic review of neoadjuvant transarterial chemoembolization for resectable hepatocellular carcinoma. Liver Int.

[7] Yamashita T, Kaneko S (2014). Orchestration of hepatocellular carcinoma development by diverse liver cancer stem cells. J Gastroenterol.

[8] Yamashita T, Wang XW (2013). Cancer stem cells in the development of liver cancer. J Clin Invest.

[9] Kim H, Choi GH, Na DC, Ahn EY, Kim GI, Lee JE, Cho JY, Yoo JE, Choi JS, Park YN (2011). Human hepatocellular carcinomas with “Stemness”-related marker expression: keratin 19 expression and a poor prognosis. Hepatology.

[10] Guo Z, Li LQ, Jiang JH, Ou C, Zeng LX, Xiang BD (2014). Cancer stem cell markers correlate with early recurrence and survival in hepatocellular carcinoma. World J Gastroenterol.

[11] Chan AW, Tong JH, Chan SL, Lai PB, To KF (2014). Expression of stemness markers (CD133 and EpCAM) in prognostication of hepatocellular carcinoma. Histopathology.

[12] Wilson GK, Tennant DA, McKeating JA (2014). Hypoxia inducible factors in liver disease and hepatocellular carcinoma: current understanding and future directions. J Hepatol.

[13] Kaur B, Khwaja FW, Severson EA, Matheny SL, Brat DJ, Van Meir EG (2005). Hypoxia and the hypoxia-inducible-factor pathway in glioma growth and angiogenesis. Neuro Oncol.

[14] Lock FE, McDonald PC, Lou Y, Serrano I, Chafe SC, Ostlund C, Aparicio S, Winum JY, Supuran CT, Dedhar S (2013). Targeting carbonic anhydrase IX depletes breast cancer stem cells within the hypoxic niche. Oncogene.

[15] Frolova O, Samudio I, Benito JM, Jacamo R, Kornblau SM, Markovic A, Schober W, Lu H, Qiu YH, Buglio D, McQueen T, Pierce S, Shpall E (2012). Regulation of HIF-1alpha signaling and chemoresistance in acute lymphocytic leukemia under hypoxic conditions of the bone marrow microenvironment. Cancer Biol Ther.

[16] Zeng Z, Ren J, O’Neil M, Zhao J, Bridges B, Cox J, Abdulkarim B, Schmitt TM, Kumer SC, Weinman SA (2012). Impact of stem cell marker expression on recurrence of TACE-treated hepatocellular carcinoma post liver transplantation. BMC Cancer.

[17] Zen C, Zen Y, Mitry RR, Corbeil D, Karbanova J, O’Grady J, Karani J, Kane P, Heaton N, Portmann BC, Quaglia A (2011). Mixed phenotype hepatocellular carcinoma after transarterial chemoembolization and liver transplantation. Liver Transpl.

[18] Sung SY, Hsieh CL, Wu D, Chung LW, Johnstone PA (2007). Tumor microenvironment promotes cancer progression, metastasis, and therapeutic resistance. Curr Probl Cancer.

[19] Kato S, Hayakawa Y, Sakurai H, Saiki I, Yokoyama S (2014). Mesenchymal-transitioned cancer cells instigate the invasion of epithelial cancer cells through secretion of WNT3 and WNT5B. Cancer Sci.

[20] Li H, Fan X, Houghton J (2007). Tumor microenvironment: the role of the tumor stroma in cancer. J Cell Biochem.

[21] Vannucci L (2015). Stroma as an Active Player in the Development of the Tumor Microenvironment. Cancer Microenviron.

[22] Xing F, Saidou J, Watabe K (2010). Cancer associated fibroblasts (CAFs) in tumor microenvironment. Front Biosci (Landmark Ed).

[23] Orimo A, Gupta PB, Sgroi DC, Arenzana-Seisdedos F, Delaunay T, Naeem R, Carey VJ, Richardson AL, Weinberg RA (2005). Stromal fibroblasts present in invasive human breast carcinomas promote tumor growth and angiogenesis through elevated SDF-1/CXCL12 secretion. Cell.

[24] Hwang RF, Moore T, Arumugam T, Ramachandran V, Amos KD, Rivera A, Ji B, Evans DB, Logsdon CD (2008). Cancer-associated stromal fibroblasts promote pancreatic tumor progression. Cancer Res.

[25] Gout S, Huot J (2008). Role of cancer microenvironment in metastasis: focus on colon cancer. Cancer Microenviron.

[26] Luo D, Wang Z, Wu J, Jiang C, Wu J (2014). The role of hypoxia inducible factor-1 in hepatocellular carcinoma. Biomed Res Int.

[27] Wykoff CC, Beasley NJ, Watson PH, Turner KJ, Pastorek J, Sibtain A, Wilson GD, Turley H, Talks KL, Maxwell PH, Pugh CW, Ratcliffe PJ, Harris AL (2000). Hypoxia-inducible expression of tumor-associated carbonic anhydrases. Cancer Res.

[28] Kaluz S, Kaluzova M, Liao SY, Lerman M, Stanbridge EJ (2009). Transcriptional control of the tumor- and hypoxia-marker carbonic anhydrase 9: A one transcription factor (HIF-1) show?. Biochim Biophys Acta.

[29] Vordermark D, Kaffer A, Riedl S, Katzer A, Flentje M (2005). Characterization of carbonic anhydrase IX (CA IX) as an endogenous marker of chronic hypoxia in live human tumor cells. Int J Radiat Oncol Biol Phys.

[30] Liu K, Min XL, Peng J, Yang K, Yang L, Zhang XM (2016). The Changes of HIF-1alpha and VEGF Expression After TACE in Patients With Hepatocellular Carcinoma. J Clin Med Res.

[31] Woelber L, Kress K, Kersten JF, Choschzick M, Kilic E, Herwig U, Lindner C, Schwarz J, Jaenicke F, Mahner S, Milde-Langosch K, Mueller V, Ihnen M (2011). Carbonic anhydrase IX in tumor tissue and sera of patients with primary cervical cancer. BMC Cancer.

[32] Zheng SS, Chen XH, Yin X, Zhang BH (2013). Prognostic significance of HIF-1alpha expression in hepatocellular carcinoma: a meta-analysis. PLoS One.

[33] Rhee H, Nahm JH, Kim H, Choi GH, Yoo JE, Lee HS, Koh MJ, Park YN (2016). Poor outcome of hepatocellular carcinoma with stemness marker under hypoxia: resistance to transarterial chemoembolization. Mod Pathol.

[34] Villanueva A, Hoshida Y, Battiston C, Tovar V, Sia D, Alsinet C, Cornella H, Liberzon A, Kobayashi M, Kumada H, Thung SN, Bruix J, Newell P (2011). Combining clinical, pathology, and gene expression data to predict recurrence of hepatocellular carcinoma. Gastroenterology.

[35] Fiaschi T, Giannoni E, Taddei ML, Cirri P, Marini A, Pintus G, Nativi C, Richichi B, Scozzafava A, Carta F, Torre E, Supuran CT, Chiarugi P (2013). Carbonic anhydrase IX from cancer-associated fibroblasts drives epithelial-mesenchymal transition in prostate carcinoma cells. Cell Cycle.

[36] Sciarra A, Ronot M, Di Tommaso L, Raschioni C, Castera L, Belghiti J, Bedossa P, Vilgrain V, Roncalli M, Paradis V (2015). TRIP: a pathological score for transarterial chemoembolization resistance individualized prediction in hepatocellular carcinoma. Liver Int.

[37] Seok JY, Na DC, Woo HG, Roncalli M, Kwon SM, Yoo JE, Ahn EY, Kim GI, Choi JS, Kim YB, Park YN (2012). A fibrous stromal component in hepatocellular carcinoma reveals a cholangiocarcinoma-like gene expression trait and epithelial-mesenchymal transition. Hepatology.

[38] Wan S, Zhao E, Kryczek I, Vatan L, Sadovskaya A, Ludema G, Simeone DM, Zou W, Welling TH (2014). Tumor-associated macrophages produce interleukin 6 and signal via STAT3 to promote expansion of human hepatocellular carcinoma stem cells. Gastroenterology.

[39] Fan QM, Jing YY, Yu GF, Kou XR, Ye F, Gao L, Li R, Zhao QD, Yang Y, Lu ZH, Wei LX (2014). Tumor-associated macrophages promote cancer stem cell-like properties via transforming growth factor-beta1-induced epithelial-mesenchymal transition in hepatocellular carcinoma. Cancer Lett.

[40] Henry LR, Lee HO, Lee JS, Klein-Szanto A, Watts P, Ross EA, Chen WT, Cheng JD (2007). Clinical implications of fibroblast activation protein in patients with colon cancer. Clin Cancer Res.

[41] Cohen SJ, Alpaugh RK, Palazzo I, Meropol NJ, Rogatko A, Xu Z, Hoffman JP, Weiner LM, Cheng JD (2008). Fibroblast activation protein and its relationship to clinical outcome in pancreatic adenocarcinoma. Pancreas.

[42] van Zijl F, Zulehner G, Petz M, Schneller D, Kornauth C, Hau M, Machat G, Grubinger M, Huber H, Mikulits W (2009). Epithelial-mesenchymal transition in hepatocellular carcinoma. Future Oncol.

[43] Won C, Kim BH, Yi EH, Choi KJ, Kim EK, Jeong JM, Lee JH, Jang JJ, Yoon JH, Jeong WI, Park IC, Kim TW, Bae SS (2015). Signal transducer and activator of transcription 3-mediated CD133 up-regulation contributes to promotion of hepatocellular carcinoma. Hepatology.

